# A Résumé of Cases Treated According to Lister’s Antiseptic Method, in Cook County Hospital

**Published:** 1879-05

**Authors:** A. Goldspohn

**Affiliations:** Formerly House Surgeon


### ARTICLE VI.

### A RÉSUMÉ OF CASES TREATED ACCORDING TO LISTER'S ANTISEPTIC METHOD IN COOK COUNTY HOSPITAL. By A. GOLDSPOHN, M.D., formerly House Surgeon.

This system of surgery was introduced in Chicago, and in this hospital during March, 1878, by Dr. Edwin Powell, who had observed its use at length in the hands of its author, Joseph Lister, at Kings College Hospital, London.

The following cases are such as occurred in a certain length of time and are taken without selection. The first nine were in care of Dr. Edwin Powell, the next two in care of Dr. E. W. Lee and the last four belonged to Prof. Moses Gunn.

*Case* 1.—P. W., æt. 39. Dry gangrene of index finger; amputation through the continuity of first phalanx. Was redressed once. Union by first intention was perfect, and only a little serous discharge was found on the dressing.

*Case* 2.—F. W., æt. 18. Abscess beneath the palmar fascia of the hand. Was lanced and considerable inspissated pus evacuated. The cavity was rendered aseptic by thorough washing with a 20 per cent. solution of carbolic acid, and dressed under the spray with the antiseptic gauze. It was redressed only a few times. The wound healed not so rapidly, but kindly and without suppuration.

(In the next case, it will be observed, the chances for infection were given so long before the Lister dressing was applied that it was scarcely any fair test).

*Case* 3.—C. K., æt 35. A compound fracture of the skull with removal of pieces from both tables at the bottom of a gaping scalp wound. A triangular excavated wound of about 15 square centimeters in area, in the right supra-clavicular region, showing the pulsations of the sub-clavian artery plainly. Also, amputation of the little, ring and middle fingers of one hand, with their metacarpal bones at and near the carpo-metacarpal joints. The surface open with no flaps. Was admitted into the hospital in this condition, 24 hours after receipt of injuries. The wounds were dressed for nine days with boracic acid, i.e., crystals and solution on lint. The hand then looked well. During this time the temperature ranged from 37 4-5° C. to 39½° C. On the 10th day the antiseptic gauze was applied to the head and the hand, and was renewed on the 11th, 12th, 13th and 14th days. The patient's course continued favorable until the 13th day, when loss of appetite, thirst and chilly sensations appeared. Subsequently severe chills occurred irregularly; the temperature rose at different times to over 40 3-5° C.; the wounds which now bore simple dressings, began to assume a shriveled appearance; and the patient failed rapidly and died on the 17th day.

*Case* 4.—J. C., æt 42. Amputation of finger, done and dressed under the carbolized spray. The dressing was renewed on the 3d day, when a little thin, reddish discharge was noticed, but no bad odor. Again, on the 12th day, the stump was substantially healed, excepting a little superficial granulating surface; but on the 22d day a collection of pus was found beneath the previously united flaps.

*Case* 5.—J. F., æt 22. Resection of elbow after destructive inflammation of the joint, following gunshot injury. A longitudinal incision at least 12 centimeters in length, was made on the dorsal aspect of the joint. The articular surfaces being destroyed, the adjoining ends of the humerus, radius and ulna were removed. The ball was found lodging in the greater sigmoid notch. No ligatures were required. A drainage tube was inserted and the wound closed with carbolized silk sutures. The operation was performed and the regular Lister dressing applied carefully under the spray. With this the arm was then adjusted upon an angular anterior splint at an angle of 45°. A small amount of anodyne was needed just after the operation, but subsequently the patient was entirely comfortable and ate three good meals daily. On the 3d day, the wound was redressed the first time. Immediate union was in progress nearly everywhere. Some dark red and watery discharge was found on the dressing, but the odor was very good. Union by first intention did occur in this case in every portion except one fistulous opening where the drainage tube had been. The wound was redressed in all five times, and at intervals of four and five days. At no time after the operation in this case, was there any marked general disturbance. The temperature ranged between 36 4-5° and 37 2-5° C., although on the evening of the 2d, 6th and 7th days, after some excitement from visitors, it was 37 3-5° C. During the whole time the patient who previously looked pale and a little emaciated, gained perceptibly in nutrition during the Lister régime. When the wound was healed, Dr. Powell had the patient anæsthetized again, and then put the forearm through its motions. These the patient was instructed to maintain, and several months later he reappeared to exhibit the arm with flexion and extension at the elbow nearly perfect.

*Case* 6.—H. S. H., æt 40. Scirrhus of female breast, amputation. Catgut ligatures were used; a drainage tube was inserted; and the wound closed by interrupted sutures and dressed antiseptically. In this case we must rely chiefly on the hospital record of the case, which is quite incomplete, except as to temperatures taken. It is known, however, that the wound healed very kindly throughout, and mostly by first intention. Some pus was noticed at several dressings; but no surgical fever of any moment occurred. The temperature rose twice to 38 3-5° C., but otherwise ranged between 37° C. and 37 4-5° C.

*Case* 7.—Mrs. S. F., æt 56. Epithelioma of forearm 4½ centimeters in diameter. Removed by oval incision, and the margins of the wound approximated with interrupted sutures. The peculiar dark serous discharge was abundant in this case. Consequently the wound was redressed after 24 hours for the first time, and afterwards three times more at intervals of two and three days. The wound healed by first intention except where the discharge from the interior made its exit. No pus was formed and no fever experienced, but the patient had some pain.

*Case* 8.—S. T., æt 45. A lacerated wound of the scalp semicircular in outline, and fully 8 centimeters in extent. Also a lacerated wound of hand, with fracture of first phalanx of second finger. Four sutures were put into the former and one into the latter wound, and both dressed antiseptically. They were redressed once i. e. on the second day ; and on the third the wounds were closed so that the patient went out at his own request. The temperature was about normal in the morning, but it rose to 39° C. and 39 1-5° C. on the evening of the first and second days respectively.

*Case* 9.—J. C.. æt 55. Encephaloid tumor of the thyroid body. The irregularly globular tumor was about 8 centimeters in diameter, and compressed the trachea considerably, so that the patient experienced marked difficulty in breathing when lying on his back. Dr. Powell made a straight incision 8 centimeters long, parallel to the inner border of the sterno-cleido-mastoid, passed his finger around the tumor, tore loose many of its attachments and removed it. Much hæmorrhage attended the operation, and several ligatures were required. The margins of the wound were stitched together and the regular antiseptic dressing applied. In this case there was a large discharge of sero-sanguinolent fluid which made it necessary to renew the dressing daily and sometimes oftener. But no pus was formed nor any bad odor observed. The temperature was 38 4-5° C. on the morning after operation ; but after that it did not exceed 38 2-5° C. The patient was always comfortable and slept well, but did not eat much. In a week after the operation the wound was nearly healed, and the patient went home of his own accord.

*Case* 10.—Lacerated wound on the dorsum of a hand, caused by a saw, cutting off the head of the metacarpal bone of the index finger, injuring the base of the first phalanx of the middle finger, and laying open the joint between the first and second phalanges of the little finger. The index finger remained attached by a palmar flap, through which some flexion was retained when the joint was fixed. The injured ends of bone and numerous strands of soft tissues were removed. The wound was carefully cleansed with 20 per cent. solution of carbolic acid under the spray, its surfaces approximated and its edges held in opposition by plastic pins. The Lister protection and gauze were applied and the hand then adjusted on a palmar splint. Was redressed on the 4th day. Union by first intention of nearly the entire surface was evident. No pus nor foul odor. Everything was in the same excellent condition on the 7th day. Healing was complete except two small granulating orifices at angles of the wound. Removed the suture pins. A simple dressing would now have been sufficient; but the gauze was put on four days more. Then the healing was perfect.

*Case* 11.—J. H., æt 29. Amputation of index finger at carpo-metacarpal joint after a suppurative lymphangitis of the hand, following a bite of the finger implicated. Was redressed after the 3d day. In the meantime the temperature was high. But it lowered after the first dressing and remained nearly normal. The amount of pus, considerable at first, became less and less with each of the following dressings which were applied on on the 6th, 7th and 10th days. The patient's general condition became very much better. (Suppuration was to be expected in this case from the previously infected tissues).

*Case* 12.—R. M., æt 13. Amputation of 3d, 4th and 5th toes with the greater portion of their metacarpal bones. A scant plantar flap was all that could be constructed; yet the wound was closed with sutures re-enforced by adhesive straps, which relieved the tension. The dressing was renewed on the 3d and 5th days, when union by first intention in about three-fourths of the wound was assured. No sloughing occurred, but a few small gaping orifices remained at angles of the wound, from which a little purulent fluid issued. While these were granulating, the regular dressing was applied five times more at intervals of three and four days. The temperature, after the first day, was 39° C., after the second day 38 2-5° C.; but afterwards never rose above 37 4-5° C.

*Case* 13.—T. K., æt 65. Necrosis of toe, amputation through the metacarpal bone. Was done à la Lister. Patient continued comfortable. The dressing was renewed on the third day, when the wound appeared quite favorable; again on the fifth and sixth days, when a little pus was found and an offensive odor. On the fifth day the nurse reported the patient to have had a chill, though he had not felt cold. Following this, the temperature rose to over 40 3-5° C. This continued without much reduction, together with a very obtuse mental condition until death on the morning of the seventh day. Post mortem showed septicæmia.

*Case* 14.—C. G., æt 53. Amputation of irritable stump of leg. The patient continued to be very comfortable, and not the slightest odor could be detected as days passed by. The leg was redressed for the first time at the end of 94 hours, when union by first intention throughout was assured. The discharge was very small in amount and all smell was "sweet." The dressing was now left on for seven days and then discontinued, the wound being healed with the exception of two very small and superficial granulating surfaces, to which carbolized cosmoline was applied. The temperature in this case was 38½° C. a few hours after the operation, but subsequently it never exceeded 37 4-5° C.

*Case* 15.—S. M., æt 7. Railroad injury, crushing of both ankles. Three hours later Dr. Gunn amputated each leg through its middle third. Hæmorrhage had been considerable before the operation; and after it the little boy was ghastly pale with scarcely any radial pulse. Reaction came slowly; and later a period of traumatic delirium was encountered. The stumps were redressed on alternate days. The proper sero-sanguineous discharge continued with a good odor of the parts until the points of the antero-posterior flaps began to slough, as they did seemingly from too great tension. Union by first intention had taken place very satisfactorily on the interior of the wounds between the bodies of the flaps; and the granulating surfaces which remained when their dead extremities had dropped off, assumed almost perfectly the condition of a typically healthy ulcer under this dressing. Before the sloughing no pus occurred, but during its continuance pus was always found on the dressings which were then changed daily. The temperature was high in this case, especially during the first week. Then it lowered and afterwards rose once more for a very short time.

REMARKS.—In order to succeed with with the Lister régime the following points should be observed:

(1.) All hæmorrhage should have ceased before closing the wound.

(2.) Animal ligatures only should be used and they should be cut short.

(3.) When a wound is large or when from its vascularity much discharge may be expected, one or more drainage tubes should be inserted, whose orifices must not be occluded by the oiled silk protective.

(4.) The entire dressing should be soaked in carbolized water, when ready cut, in order to make it more pliable. This feature is desirable not merely for the greater comfort it affords the patient, but especially because the individual pieces can be more exactly molded to the surfaces adjacent to the wound.

The conditions from which we recognize the efficiency of a dressing or the necessity for its renewal are (1), The odor of the part (2), The amount and character of the discharge, and (3), Pain and temperature. If the odor becomes putrid; if the discharge becomes purulent or so great in amount as to moisten the outermost layers of the dressing: if pain be unduly great; if the temperature be very high or take a sudden rise; if any one or more of these features exist, a renewal is called for; but in their absence the same dressing may be left on indefinitely.

